# 3D-2D Deformable Image Registration Using Feature-Based Nonuniform Meshes

**DOI:** 10.1155/2016/4382854

**Published:** 2016-02-25

**Authors:** Zichun Zhong, Xiaohu Guo, Yiqi Cai, Yin Yang, Jing Wang, Xun Jia, Weihua Mao

**Affiliations:** ^1^University of Texas at Dallas, Richardson, TX 75080, USA; ^2^University of Texas Southwestern Medical Center, Dallas, TX 75235, USA; ^3^University of New Mexico, Albuquerque, NM 87131, USA

## Abstract

By using prior information of planning CT images and feature-based nonuniform meshes, this paper demonstrates that volumetric images can be efficiently registered with a very small portion of 2D projection images of a Cone-Beam Computed Tomography (CBCT) scan. After a density field is computed based on the extracted feature edges from planning CT images, nonuniform tetrahedral meshes will be automatically generated to better characterize the image features according to the density field; that is, finer meshes are generated for features. The displacement vector fields (DVFs) are specified at the mesh vertices to drive the deformation of original CT images. Digitally reconstructed radiographs (DRRs) of the deformed anatomy are generated and compared with corresponding 2D projections. DVFs are optimized to minimize the objective function including differences between DRRs and projections and the regularity. To further accelerate the above 3D-2D registration, a procedure to obtain good initial deformations by deforming the volume surface to match 2D body boundary on projections has been developed. This complete method is evaluated quantitatively by using several digital phantoms and data from head and neck cancer patients. The feature-based nonuniform meshing method leads to better results than either uniform orthogonal grid or uniform tetrahedral meshes.

## 1. Introduction

Cone-Beam Computed Tomography (CBCT) has been widely used for accurate patient setup (initial positioning) and adaptive radiation therapy. Attentions are still needed to reduce imaging radiation doses and improve image qualities. Traditionally, Conventional Cone-Beam Computed Tomography (CBCT) image reconstruction in radiation therapy needs hundreds of projections, which deliver high imaging dose to patients. In order to reduce the number of CBCT projections, recently, some researchers have proposed methods to reconstruct images by using the information of prior images, such as a planning CT [1–3] or a previous CBCT [4], and a deformation model, which is essentially a 3D-2D deformable image registration (DIR) procedure. A lot of researches have been done related to 3D-2D image registration. Previously, only rigid registrations between 3D images and 2D fluoroscopic images have been addressed [5–7]. For 3D-2D nonrigid registration [1, 2, 4, 8, 9], the multiscale technique was applied, instead of using the finite element method- (FEM-) based methods to speed up the reconstruction process and increase the accuracy. For nonrigid modeling of respiratory motion, Zeng et al. [10] introduced a method to estimate the 3D motion parameters of a nonrigid, free breathing motion model from a set of projection views. In order to improve the computational efficiency, Jia et al. [11] developed a GPU-based algorithm to reconstruct high-quality CBCT images from undersampled and noisy projection data so as to lower the imaging dose, but it does not make use of the planning CT. However, in all these methods, voxel-based deformation fields were employed to estimate a large number of unknowns, which required extremely long computational time. Additionally, in their deformation models, the image features and organ boundaries were not specifically considered, which may cause inaccurate deformation estimation. In this work, we proposed a new FEM-based approach, that is, a feature-based nonuniform meshing method, to overcome these limitations.

FEM can be best understood from its practical application, for instance, mesh discretization of a continuous domain into a set of discrete subdomains. It has already been used in image registration [12, 13]. Usually, FEM is exploited to achieve two important advantageous aspects. On one hand, it endows the efficiency of the registration process due to a small number of sampling points compared with voxel-based sampling methods. On the other hand, it provides the smoothness of the displacement vector field (DVF) due to the smoothness constraint between elements and the interpolation within one element. The quality of geometric discretization is crucial for the effectiveness of the image registration applications. Surface meshing methods in 3D-3D image registration [13–15] and volume meshing methods in 3D-3D image registration [16–19] and in 3D image reconstruction [20, 21] have been applied, but none of them were employed in 3D-2D DIR.

When 3D-2D DIR algorithm is used iteratively to reconstruct 3D volumetric images, the number of sampling points is crucial for the computation. A large number of sampling points could lead to a very slow computational speed, while a limited number of points with uniform distribution could miss some important image features and make the registration less accurate. In our proposed method, a special FEM system is developed to automatically generate high-quality adaptive meshes conforming to the image features for the whole volume without user's manual segmentation. This system allows for more sampling points placed in important regions (at organ/tissue/body boundaries or regions with highly nonlinearly varying image intensity); while fewer sampling points are placed within homogeneous or in regions with linearly varying intensity. In this way, deformations of boundaries and other important features can be directly characterized by the displacements of the sampling points that are lying on boundaries or features, rather than interpolating from a uniform grid or a larger-sized tetrahedron in the volume mesh. As a result, the deformation can be controlled more precisely. With approximately the same numbers of sampling points, the feature-based nonuniform meshing method produces better deformed volumetric images comparing with methods using uniform meshes. The high-quality digitally reconstructed radiographs (DRRs) of the deformed anatomy are generated by using ray tracing method. Subsequently, these DRRs are compared with corresponding 2D projections from CBCT scans, and the DVF is optimized iteratively to obtain the final reconstructed volumetric images.

In order to provide a good initial DVF and accelerate the calculation, we proposed a boundary-based 3D-2D DIR method before the aforementioned 3D-2D DIR. Although researches on boundary-guided (or contour-guided) image registrations [22–24] have been carried out for many years, their methods were applied on either 2D-2D or 3D-3D DIR cases. Our proposed algorithm is suitable to employ on 3D-2D DIR, while dealing with large deformations for adaptive radiation therapy.

This paper makes the following contributions for effectively computing 3D-2D DIR:

- Compared with the traditional voxel-based method, the mesh-based methods have faster computational speed and better DVFs, since the voxel-based deformations were employed to estimate a large number of unknowns, which required extremely long computational time and were easy to be trapped in localized deformations.
- When equal numbers of sampling points are used, the nonuniform meshing method leads to obtaining higher quality reconstructed images and better DVF compared with that of uniform meshes under the same number of optimization iterations.
- Due to the large data sizes of the volume and projection images, the boundary-based DIR technique and GPU-based parallel implementation have been applied and achieved high computational efficiency and the reconstruction of 512 × 512 × 140 CBCT image can be done within 3 minutes, which is close to clinical applications.

The rest of the paper is organized as follows.  Section 2 describes the proposed methods and materials in detail, including nonuniform mesh generation, the framework of the nonuniform meshing to reconstruct volumetric images by using 3D-2D DIR, and the boundary-based 3D-2D DIR. In Section 3, the experimental results are discussed to evaluate the proposed methods qualitatively and quantitatively. Several digital phantoms and patient data sets are measured. Finally, the conclusion and future work are given in Section 4.

## 2. Methods and Materials

**Figure 1:**
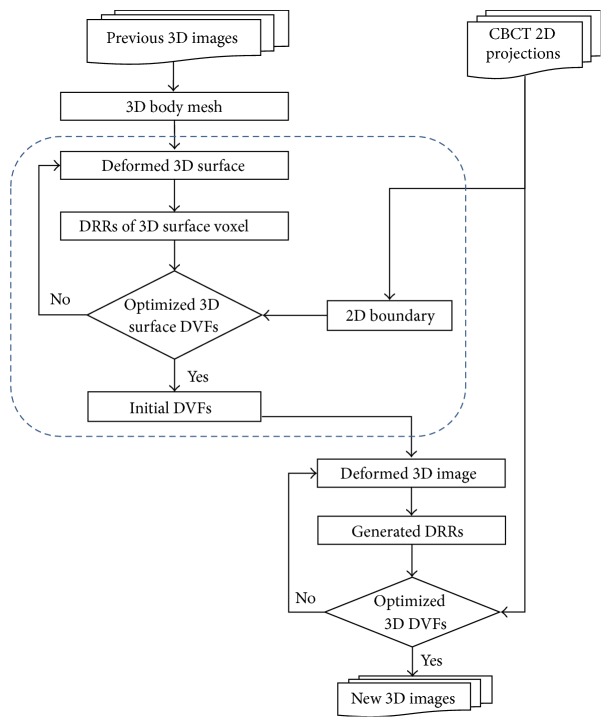
Flow chart of the proposed 3D-2D image registration method. The dash-line box is used to provide initial DVFs and accelerate the calculation by proposed boundary-based 3D-2D DIR method. It may be skipped according to different scenarios. More details are given in Section 2.3.

Figure 1 illustrates a flow chart of the entire proposed technique. The dash-line box is used to provide initial DVFs and accelerate the calculation by proposed boundary-based 3D-2D DIR method, which will be described in Section 2.3. All other parts illustrate how the proposed novel nonuniform-mesh-guided 3D-2D image registration method is used to deform the original planning CT images. This requires much smaller degrees of freedom to generate the DVF than that of voxel-based methods. Each step is described in the following subsections and we will firstly introduce the nonuniform-mesh-guided 3D-2D image registration method and then discuss the boundary-based 3D-2D DIR method.

### 2.1. Creation of Nonuniform Meshes

After the user specifies the total number of mesh vertices, a feature-based nonuniform tetrahedral mesh is generated automatically. Nonuniform meshes are important for improving the accuracy of the numerical simulations as well as better approximating the shapes. Zhong et al. [25] developed a novel particle-based nonuniform surface meshing approach by formulating the interparticle energy optimization in a fast convergence technique. In this method, users will design a density field, which is used to control the distribution of the particles (sampling points). This particle-based surface meshing framework is extended to 3D volume case in this paper so as to generate tetrahedral meshes based on 3D volume images.

#### 2.1.1. Basic Meshing Framework

The basic idea of the mesh generation is similar to Zhong et al.'s work [25], and the main difference is that we extend their work to 3D nonuniform volume meshing. There are two steps in mesh generation: particle optimization and Delaunay triangulation computation.

Regarding each mesh vertex as a particle, the potential energy between the particles decides the interparticle forces. When the forces on each particle reach equilibrium, particles arrive at an optimal balanced state, resulting in a uniform distribution. In this case, an isotropic meshing can be generated. To handle the adaptive meshing, the concept of “embedding space” [26, 27] is applied. In the Nash embedding theorem, it is stated that every Riemannian manifold [28] can be isometrically embedded into some high-dimensional Euclidean space. In such high-dimensional embedding space, the metric is uniform and isotropic. When the forces applied on each particle become equilibrial in this embedding space, the particle distribution in the original domain will exhibit the desired adaptive property, that is, conforming to the user-defined density field. This property is used to formulate the particle-based adaptive meshing framework. The following concepts of density field, interparticle energy, and force are defined based on [25].

The density field is defined by using the following metric tensor as

(1) $$\begin{array}{c} M\left(\;v\;\right)=\rho {\left(\;v\;\right)}^{2/m}\cdot I, \end{array}$$

where **v** is the particle position. **I** is the 3 × 3 identity matrix. **M**(**v**) defines an isotropic adaptive metric with the user-defined density function *ρ*(**v**). *m* is the dimension of the original volume space, so *m* = 3.

Given *N*_*v*_ particles with their positions **V** = {**v**_*i*_∣*i* = 1 ⋯ *N*_*v*_} in the volume *Ω* (*Ω* ∈ *ℝ* ^*m*^) which is embedded in $R^{\overset{-}{m}}$ space, denoted as $\overset{-}{V}=\left\{{\overset{-}{v}}_{i}|i=1\cdots N_{v}\right\}$, where $m\leq \overset{-}{m}$, the interparticle* energy* between particles *i* and *j* in such embedding space is defined as

(2) $$\begin{array}{c} {\overset{-}{E}}^{ij}=e^{-{\left\|{\overset{-}{v}}_{i}-{\overset{-}{v}}_{j}\right\|}^{2}/4\sigma^{2}}=e^{-{\left(v_{i}-v_{j}\right)}^{T}M_{ij}\left(v_{i}-v_{j}\right)/4\sigma^{2}}, \end{array}$$

where **M**_*ij*_ is the metric tensor between particles *i* and *j*, and, for simplicity, it is approximated by the average of metric tensors at two positions: **M**_*ij*_ ≈ (**M**(**v**_*i*_) + **M**(**v**_*j*_))/2. The exponent in the term ${\overset{-}{E}}^{ij}$ can be approximated by ${\left\|{\overset{-}{v}}_{i}-{\overset{-}{v}}_{j}\right\|}^{2}\approx {\left(v_{i}-v_{j}\right)}^{T}M_{ij}(v_{i}-v_{j})$. The interparticle energy as defined in (2) depends on how to choose the fixed kernel width *σ*. If *σ* is chosen too small, then particles will nearly stop spreading because there are almost no forces between particles. If *σ* is chosen too large, then nearby particles cannot repel each other and the resulting sampling distribution will be poor. From our extensive experiments, we find out that the best adaptive mesh quality can be achieved when Gaussian kernel width is set as $\sigma =0.3\sqrt{\left|\overset{-}{\Omega }\right|/N_{v}}$. $\left|\overset{-}{\Omega }\right|$ is the image volume in the embedding space.

The total energy can be computed by summing up all pairs of interparticle energies:

(3) $$\begin{array}{c} \overset{-}{E}=\overset{N_{v}}{\underset{i=1}{\sum }}\;\overset{N_{v}}{\underset{j=1,j\neq i}{\sum }}{\overset{-}{E}}^{ij}. \end{array}$$

The gradient of ${\overset{-}{E}}^{ij}$ can be considered as the* force* ${\overset{-}{F}}^{ij}$ in the embedding space:

(4) F−ij∂E−ij∂v−j=v−i−v−j2σ2e−v−i−v−j2/4σ2=Qijvi−vj2σ2e−vi−vjTMijvi−vj/4σ2,

where $Q_{ij}=\sqrt{M_{ij}}$. The details of the mathematical derivations are given in Sec. 3.2.2 of [25].

Then the total force applied on particle *i* is

(5) $$\begin{array}{c} {\overset{-}{F}}^{i}=\underset{j\neq i}{\sum }{\overset{-}{F}}^{ji}. \end{array}$$

In the particle optimization algorithm, user can specify a density field *ρ*(**v**) and desired number of vertices *N*_*v*_. In our implementation, for each particle, we only compute the mutual effects (i.e., energy and forces) from the particles within a neighborhood of five standard deviations (5*σ*); otherwise, the particles have no mutual effects due to the large distance between each other. The *k*-*d* tree [29] is a space-partitioning data structure for organizing points in a *k*-dimensional space and can quickly search such neighborhoods. With the total interparticle energy (3) and force (5), L-BFGS [30] (a quasi-Newton algorithm) optimization method is used to obtain the optimized adaptive particle positions. This optimization proceeds iteratively until convergence by satisfying a specified stopping condition; for example, the magnitude of the gradient or the maximal displacement of particles is smaller than a threshold, or the total number of iterations.  Algorithm 1 shows the details of the adaptive particle optimization on 3D volume.

After optimizing the particle positions, the final desired nonuniform tetrahedral mesh can be generated by using the Delaunay triangulation [29]. If the density field is uniform in the entire volume, we can generate the isotropic tetrahedron mesh, which is used in Section 3 for comparison experiments.

#### 2.1.2. Feature-Based Nonuniform Meshing on Medical Image

**Figure 2:**
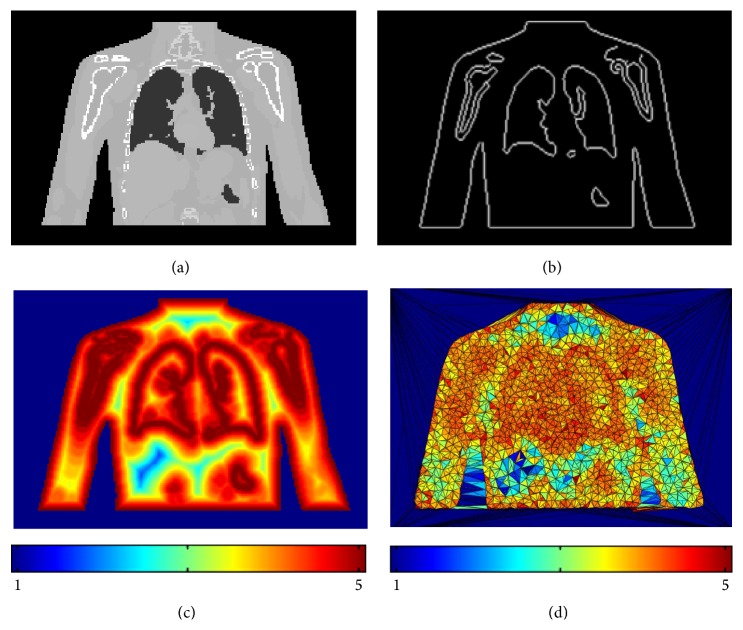
Demonstration of the feature-based nonuniform mesh generation on a digital XCAT phantom. (a) The original image; (b) extracted feature edges; (c) density field; (d) a 2D view of the interior meshes with color-mapping.

Figure 2 illustrates the feature-based nonuniform mesh generation on a set of torso images acquired from a digital phantom XCAT [31]. The 4D XCAT provides an accurate representation of complex human anatomy and has the advantage that its organ shapes can be changed to realistically model anatomical variations and patient motions; more importantly, it also provides voxel-based DVFs, which are used as the ground truths to evaluate the accuracy of the deformation.

It is necessary to design a density field to match the volume image features. Original images are preanalyzed using a Laplacian operator (searching for zero crossings in the second derivative of the image to find edges) to extract features including contour edges and boundaries between organs and tissues, which are regions with highly nonlinearly varying image intensities. Since the Laplacian operator as high-pass operator highlights edges as well as noise, it is desirable to smooth the image in order to suppress noises at first. When the feature edges of the volume image are obtained as shown in Figure 2(b), a density field could be calculated automatically without manual segmentation (Figure 2(c)). The density function *ρ*(**v**)^1/3^ depends on the distance between the feature edges and the voxels in the volume domain. In our experiments, the smooth density field is defined as a piecewise linear function:

(6) $$\begin{array}{c} \rho {\left(\;v\;\right)}^{1/3}=\left\{\begin{array}{cc} 5, & 0\leq \varphi \leq 2,\\ \frac{49}{9}-\frac{2}{9}\varphi , & 2<\varphi \leq 20,\\ 1, & \text{otherwise}, \end{array}\right. \end{array}$$

where *φ* is the distance between the feature edges and the voxels measured by the voxel grid unit. The red color means the higher density field area, while the blue color means the lower density field area. The user can choose any other density functions according to their requirement. The motivation of designing the density field as a piecewise linear function is to make the density field as smooth as possible, so that the volume sizes of the tetrahedrons in the computed mesh can be controlled to vary smoothly. Finally, we can generate adaptive meshes with high-quality tetrahedral elements. Equation (6) is given under our extensive DIR experiments, which is based on the volume image resolutions and voxel scales: if the sampling point is located within 2-voxel distance with respect to its nearest feature edge point, the density value is 5; if the sampling point is located between 2-voxel and 20-voxel distance with respect to its nearest feature edge point, the density value is computed based on the designed linear function in (6); if the sampling point is located beyond 20-voxel distance with respect to its nearest feature edge point, the density value is 1. From Figure 2, we can see that the designed density field can generate good tetrahedral meshes, which can well conform to the image features as well as obtain high-quality tetrahedral meshes.

**Figure 3:**
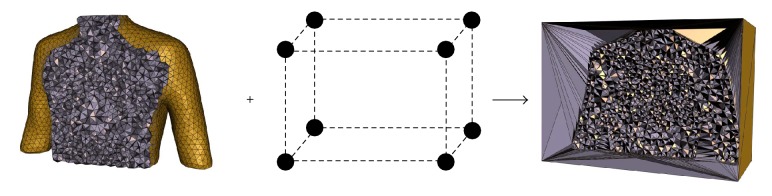
In order to control the entire volume image more effectively, 8 bounding box vertices of the human anatomy are added.

After designing the density field, a binary mask needs to be computed from the original image by setting “one” inside of the human anatomy and “zero” outside to constrain the vertex positions inside or on the body during mesh vertices optimization. The mesh vertices are automatically computed by Algorithm 1. Vertices are densely positioned in the regions with highly nonlinearly varying image intensities, while regions of constant or linearly varying image intensities are assigned fewer vertices. Following this process, vertex locations are optimized conforming to the density field as illustrated in Figure 2(c). In order to control the entire volume image more effectively, 8 bounding box vertices of the human anatomy are added (as shown in Figure 3). Then volume meshes (tetrahedrons) are created based on the Delaunay triangulation of the vertices. As a result, meshes corresponding to boundaries between organs and tissues are denser. The color-coded tetrahedrons of the generated feature-based mesh in Figure 2(d) illustrate that the tetrahedral volumes are well conforming to the desired density defined by the features of the given image. Due to the importance of deformations occurring around boundaries between organs and tissues, it is necessary to place more vertices (or sampling points) at features, while placing fewer vertices in nonfeature regions. In this way, if DVF is specified to each mesh vertex (or sampling point), the boundaries and other important features can be directly represented by the displacements of sampling points or represented by smaller tetrahedrons, rather than interpolating through four vertices of one larger-sized tetrahedron; then the deformation can be diffused from the mesh vertices to each voxel of the volume more accurately. This is the most significant advantage of the feature-based nonuniform meshing method.

### 2.2. Volumetric Image Reconstruction by 3D-2D DIR

This section introduces how to use our generated feature-based nonuniform meshing to reconstruct high-quality volumetric images by using 3D-2D DIR.

#### 2.2.1. Computation of Deformed Volume

The displacement vector of each voxel (**D** ^*v*^) is obtained through interpolating the DVF on mesh vertices (**D**) by using the barycentric coordinates [32] of each voxel in its corresponding tetrahedron, and the deformed volume is resampled onto a uniform grid volume. The intensity of each voxel in the new deformed image *U*_new_ is calculated from the original CT image *U*_original_ according to **D** ^*v*^ as follows:

(7) Unewa,b,c=Uoriginala+D1va,b,c,b+D2va,b,c,c+D3va,b,c,

where *D*_1_^*v*^, *D*_2_^*v*^, and *D*_3_^*v*^ are three floating point spatial components of the displacement vector (**D** ^*v*^) along *x*, *y*, and *z* directions. The displacement vector points from the center of voxel (*a*, *b*, *c*) in the new deformed image to a point at (*a* + *D*_1_^*v*^(*a*, *b*, *c*), *b* + *D*_2_^*v*^(*a*, *b*, *c*), *c* + *D*_3_^*v*^(*a*, *b*, *c*)) in the original source image that will unlikely be at a voxel center. Then the original source intensity at that point is obtained via trilinear interpolation from its eight neighboring voxels. This technique not only gives an accurate CT intensity to map to the new deformed image, but also acts as an antialiasing technique to avoid artifacts in the projected DRRs, since it is easy and efficient to compute DRRs from a uniform grid sampled volume by using ray tracing algorithm.

#### 2.2.2. Computation of DRR Using Ray Tracing Algorithm

Ray tracing is a technique for generating an image by tracing the path of light through pixels in an image plane and simulating the effects of its encounters with objects. In the DRR generation, the Siddon ray tracing algorithm is applied [33].

To better simulate the realistic raw target CBCT projections from XCAT phantom data and test the sensitivity of our method to the realistic complications, after the noise-free ray line integrals *p*_*i*_ are computed, the noisy signal *I*_*i*_ at each pixel *i* is generated based on the following noise model:

(8) $$\begin{array}{c} I_{i}=\text{Poisson}\left(\;I_{0}e^{-p_{i}}\;\right)+\text{Normal}\left(\;0,\sigma_{e}^{2}\;\right), \end{array}$$

where Poisson is Poisson distribution and Normal is normal distribution. *I*_0_ is the incident X-ray intensity and *σ*_*e*_^2^ is the background electronic noise variance. In this study, *I*_0_ is set to 1 × 10^5^ and *σ*_*e*_^2^ is set to 10 [34, 35].

#### 2.2.3. Optimization of 3D-2D DIR Energy

The deformation is optimized by minimizing the total energy *E*, which includes two terms, the regularization (*E*_reg_(**D**)) used to achieve smoothness of the DVF and the similarity (*E*_sim_(**D**)) between the two images:

(9) EDEregD+EsimD=μLD+∑m=1NpRD,θm−Iθm2,

where *μ* is a weighting factor to control the tradeoff between the similarity and regularization. It is empirically set at 10.0 for all of the experiments in this paper. **D** is the DVF defined on the mesh vertices. *L*(**D**) is regularization term defined as

(10) $$\begin{array}{c} L\left(\;D\;\right)=\overset{N_{v}}{\underset{i=1}{\sum }}\;\overset{3}{\underset{d=1}{\sum }}\left[\;{\left(\;\frac{\sum_{j\in N\left(i\right)}\left(D_{d}\left(j\right)-D_{d}\left(i\right)\right)}{\left|N\left(i\right)\right|}\;\right)}^{2}\;\right], \end{array}$$

where *L*(**D**) is a summation of the square of Graph Laplacian operations [36] on the DVF over every vertex except those on the external borders. **D**_*d*_ (*d* = 1,2, 3) are three components of DVFs. *N*_*v*_ is the total number of the mesh vertices. *N*(*i*) is the set of one-ring neighboring vertices (*j*) of vertex *i*. |*N*(*i*)| is the size of set *N*(*i*).

The second term of the energy function in (9) represents the similarity between the CBCT projections (*I*(*θ*_*m*_)) acquired at gantry angle (*θ*_*m*_) beforehand and the DRRs (*R*(**D**, *θ*_*m*_)) created after the deformation of **D** applied on planning CT at the same gantry angle. *N*_*p*_ is the number of projections involved. This term is a summation of the square of intensity difference over every pixel of all projections. Although *R* and *I* are two-dimensional in reality, we can easily use one-dimensional arrays to represent them for computational simplification.

L-BFGS algorithm [30] is used to optimize the DVF (**D**). The gradient of the energy function *E* with respect to **D** can be calculated as follows:

(11) ∇ED=μ∇LD+2∑m=1NpRD,θm−Iθm∇RD,θm.

For each iteration of L-BFGS optimization, the energy *E* and its gradient ∇*E* are updated.

The DRRs (*R*(**D**, *θ*_*m*_)) are generated from the resampled deformed planning CT (*U*_new_) by the ray tracing method for each gantry angle as

(12) $$\begin{array}{c} R\left(\;D,\theta_{m}\;\right)=P\left(\;\theta_{m}\;\right)U_{new}\left(\;D\;\right), \end{array}$$

where **P**(*θ*_*m*_) is the cone-beam projection matrix that describes the X-ray projection operations. The element *ω* ^*α*,*β*^ of matrix **P** is the weight of voxel *β* in *U*_new_ contributed to the pixel *α* in DRR during the projection simulation calculated by the ray tracing method.

Using (12), the gradient of the energy function *E* of (11) becomes

(13) ∇ED=μ∇LD+2∑m=1NpPθm·PθmUnewD−Iθm∇UnewD.

#### 2.2.4. GPU-Based Acceleration

The entire process of this volumetric image reconstruction method was implemented on GPU. The GPU card used in our experiments is an NVIDIA GeForce GTX 780 Ti with 3 GB GDDR5 video memory. It has 2,880 CUDA cores with a clock speed of 1,006 MHz. Utilizing such a GPU card with tremendous parallel computing ability can significantly increase the computation efficiency. There are two time-consuming processes during the reconstruction. One is the DRR generation, and the other is the gradient computation of the similarity term in the total energy *E*.

(*1) DRR Generation on GPU*. For the DRR generation part, it is straightforward to accomplish the ray tracing algorithm in parallel computation. For example, each pixel intensity of the DRR is determined by accumulating all of the weighted voxel intensities through which one X-ray goes. This computation process is highly independent between each ray line. In this case, different GPU threads can compute each ray line simultaneously without conflict. 

(*2) Computation of Energy Gradient on GPU*. From (13), it can be seen that there are two terms in the gradient of the energy. One is the gradient of regularization *μ*∇*L*(**D**) with respect to DVF **D**: this can be easily computed in parallel based on each mesh vertex. The other is the gradient of the similarity term. In order to demonstrate clearly how to compute this term in parallel, the second part of (13) is rewritten in more detail based on pixel *α* in DRR and voxel *β* in *U*_new_ at one projection in angle (*θ*_*m*_). The gradient of the similarity term with respect to displacement vector of vertex *i* (i.e., **D**_*i*_) can be denoted as follows:

(14) ∂EsimD,θm∂Di=2∑β=1UnewD,i ∑α=1Rβωα,βRα−Iα∇UnewβD,i,

where *U*_new_(**D**, *i*) is the set of voxels controlled by vertex *i*. *R*(*β*) is the set of pixels on DRR affected by voxel *β*. |*U*_new_(**D**, *i*)| and |*R*(*β*)| are the sizes of the corresponding sets. *R* ^*α*^ is the intensity of pixel *α* on DRR. *I* ^*α*^ is the intensity of pixel *α* on CBCT projection. *ω* ^*α*,*β*^ is one element in the projection matrix **P**(*θ*_*m*_), that is, the weight of voxel *β*(*U*_new_^*β*^(**D**, *i*)) contributed to the pixel *α* in DRR generation. ∇*U*_new_^*β*^(**D**, *i*) is the gradient of voxel *β*(*U*_new_^*β*^(**D**, *i*)) with respect to **D**_*i*_.

Then the gradient of the similarity term, that is, (14), can be rewritten by simplification as

(15) $$\begin{array}{c} \frac{\partial E_{sim}\left(D,\theta_{m}\right)}{\partial D_{i}}=\overset{\left|U_{new}\left(D,i\right)\right|}{\underset{\beta =1}{\sum }}\frac{\partial E_{sim}\left(D,\theta_{m}\right)}{\partial U_{new}^{\beta }\left(D,i\right)}\frac{\partial U_{new}^{\beta }\left(D,i\right)}{\partial D_{i}}. \end{array}$$

Finally the total gradient for all projections with respect to displacement vector **D**_*i*_ is

(16) $$\begin{array}{c} \frac{\partial E_{sim}\left(D\right)}{\partial D_{i}}=\overset{N_{p}}{\underset{m=1}{\sum }}\;\overset{\left|U_{new}\left(D,i\right)\right|}{\underset{\beta =1}{\sum }}\frac{\partial E_{sim}\left(D,\theta_{m},\beta \right)}{\partial D_{i}}, \end{array}$$

where *N*_*p*_ is the number of projections.

Now it is clearly shown that there are two components in the gradient of the similarity term in (15).

**Figure 4:**
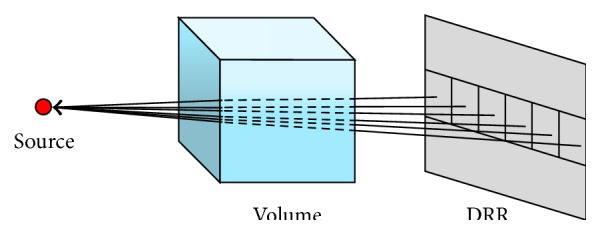
Subdividing the DRR image into small subgroups for GPU parallel computation.

(a) Gradient computation of the similarity energy with respect to voxel intensity (∂*E*_sim_(**D**, *θ*_*m*_)/∂*U*_new_^*β*^(**D**, *i*)): this can be computed when the DRRs are generated and then stored in a volume-sized matrix. However, this computation is a little bit complicated for GPU computation. One voxel of the CT image may probably affect a number of pixels on the DRR image during the projection simulation, so that in the gradient computation, it is inevitable to consider it; that is, when mapping back the DRRs to the volume image, there is probably more than one ray line going through one voxel. If the original ray tracing implementation is directly used to parallelize all ray lines in the gradient computation, there will be a memory conflict within the GPU; that is, the gradient of the similarity energy *E*_sim_ with respect to one voxel intensity is updated by different ray lines simultaneously. To overcome this problem, the DRR image is subdivided into small subgroups so that the ray lines in different groups cannot go through the same voxel concurrently as shown in Figure 4. Currently, the computation can be computed on each voxel simultaneously by assigning it to each GPU thread. After that the total gradient of the similarity term as (16) is obtained by summing up all the gradient values from different subgroups and projections.

To maximally utilize the GPU's parallel computing power, the best subgroup size for the XCAT data is 8 × 8 pixels, and the head and neck (H&N) patient data is 16 × 24 pixels, which is determined during the preprocessing step. The general idea is computed based on similar triangle property according to the voxel scale, the pixel scale, the distance from source to volume position, and the distance from source to DRR position.

(b) Gradient of voxel intensity with respect to displacement vector of vertex *i*, (∂*U*_new_^*β*^(**D**, *i*)/∂**D**_*i*_): this can be done in parallel based on each mesh vertex when the deformed volume is computed, which is independent of the projection computation.

The CPU-based serial implementation of mesh-based 3D-2D registration method on XCAT data (256 × 256 × 132) with 60 projections (256 × 256) takes about 2.5 hours; after using the GPU-based parallel implementation, it takes about 3 minutes, which is about 50 times faster.

#### 2.2.5. A Multiresolution Scheme

The size of H&N patient data used in this study is relatively large. CT volume data size is 512 × 512 × 140 and CBCT projection size is 1024 × 768. The reconstruction running time of CPU-based implementation on mesh-based 3D-2D registration method with 30 projections is about 12 hours, while the running time of GPU-based implementation is about 20 minutes, which is about 36 times faster than the CPU-based one. The multiresolution scheme is used to further improve the speed. In the experiment, both the CT volume image and CBCT images are downsampled into different resolution levels (three levels for experiments on H&N patient data), from the coarsest level (CT volume: 256 × 256 × 70, CBCT projection: 256 × 192, and time per iteration: 2.08 seconds) to the higher level (CT volume: 512 × 512 × 140, CBCT projection: 512 × 384, and time per iteration: 9.91 seconds) and finally to the full resolution level (CT volume: 512 × 512 × 140, CBCT projection: 1024 × 768, and time per iteration: 40.44 seconds). By using this strategy, the volumetric image reconstruction can be accomplished in 6.9 minutes, including 30 iterations of coarsest level, 15 iterations of higher level, and 5 iterations of full resolution level (about 60 times faster than CPU-based serial implementation). It is comparable to the fastest iterative CBCT techniques.

### 2.3. A Boundary-Based 3D-2D DIR

In order to further improve the computational speed of the proposed 3D-2D DIR method, in this section, we introduce a boundary-based 3D-2D DIR to obtain a good initial deformation; then the feature-based nonuniform meshing for 3D-2D DIR method, as mentioned in Section 2.2, is used to generate the final volumetric images.

#### 2.3.1. Extraction of 3D and 2D Boundaries

**Figure 5:**
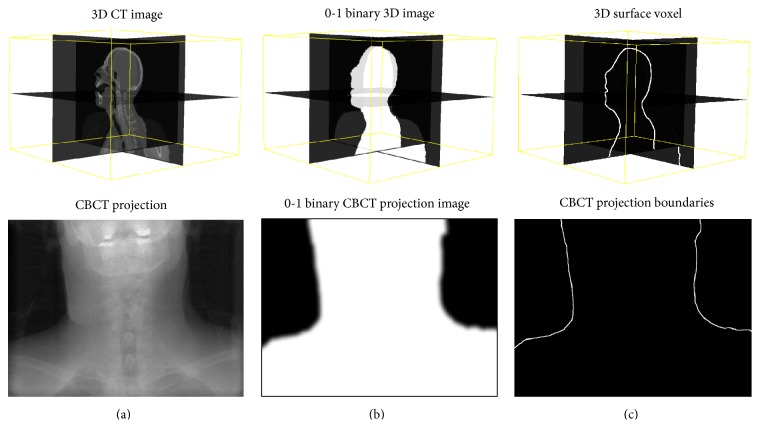
(a) Original images; (b) the 0-1 binary images; (c) boundaries of an H&N patient data.

After generating the feature-based nonuniform meshes (Section 2.1), both planning CT images and CBCT projections are preprocessed to create binary masks by setting “one” inside of the studied tissue and “zero” outside as shown in Figure 5(b). Then, the 3D tissue surface and CBCT projection boundaries are extracted by Canny edge detector [37] (Figure 5(c)).

#### 2.3.2. Computation of Projections by Splatting Method

**Figure 6:**
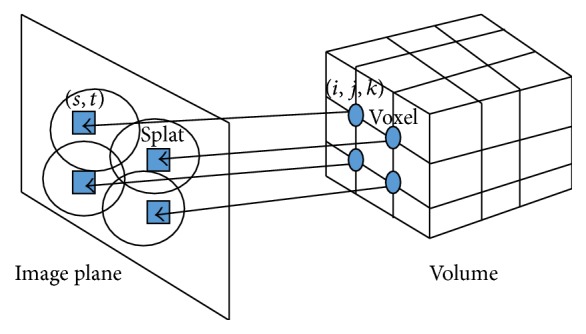
Splatting projection.

In order to directly and conveniently control the updated positions of the deformed anatomy surface voxels, we prefer to use the splatting method [38] to generate projections of 3D surface, instead of using the ray tracing method. This is one main advantage of splatting method. Every voxel's contribution to a projection is mapped directly onto the image plane by a kernel centered on the voxel as shown in Figure 6. This reconstruction kernel is called a “splat” or “footprint”:

(17) fx,y=∑i,j,kvoxeli,j,kkernelx,y=∑i,j,kvoxeli,j,k12πσ2e−x−s2/2σ2+y−t2/2σ2,

where *f*(*x*, *y*) is the final pixel intensity of the projection image and voxel(*i*, *j*, *k*) is the intensity of voxel at (*i*, *j*, *k*) position. kernel(*x*, *y*) is the “footprint function” centered at (*s*, *t*). *x* and *y* are the Gaussian kernel area within radius 3*σ* on the projection image. *σ* is the Gaussian kernel width.

**Figure 7:**
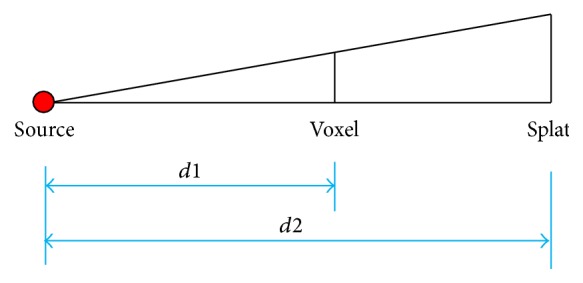
Geometry of the perspective projection with antialiasing for splatting method.

For perspective projection with antialiasing consideration, the Gaussian kernel radius should have dynamic sizes, which can be calculated from similar triangles shown in Figure 7 and

(18) d1d2=voxel scaler,σ=0.57r,

where *d*1 is the distance from the X-ray source to the voxel center and *d*2 is the distance from the source to kernel center on the image plane going through a specific voxel point. *r* is the size of splat, and voxel scale is the size of the voxel. The best coefficient value between *σ* and *r* is 0.57 based on our extensive experimental results. If the volume is a regular grid, voxel scale is fixed for all the voxels.

Another main advantage of splatting method over ray tracing is that splatting has a faster calculation speed; that is, it is very easy to ignore empty voxels (nonsurface voxels), which do not contribute to the final projection image. However, this is difficult to realize in ray tracing method.

**Figure 8:**
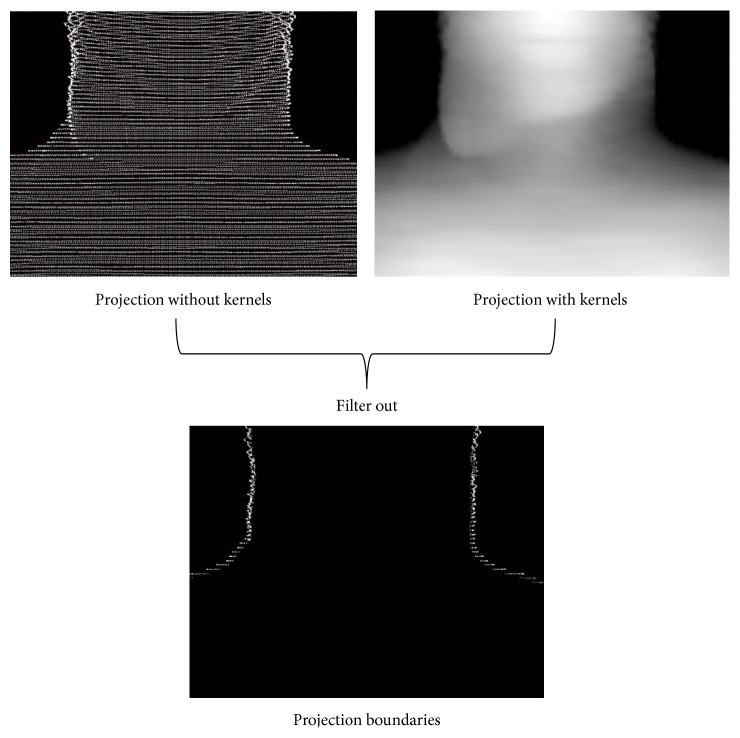
Computation of 2D projection boundaries of the deformed anatomy surface from an H&N patient data.

**Figure 9:**
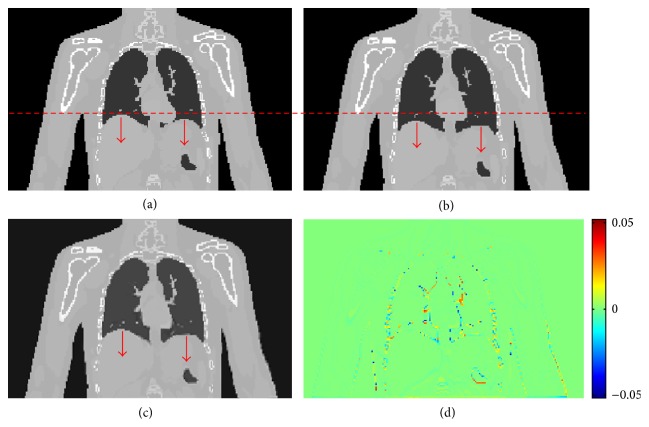
Demonstration of XCAT male phantom results. (a) Original image (Phase 1); (b) target image (Phase 4); (c) deformed image from Phase 1; (d) differences between deformed and target images.

It is noted that if we directly project the volume surface voxels (without considering kernels) onto the image plane, some pixels may be included there not belonging to the final 2D boundaries of the projection as shown at the top left of Figure 8. At the same time, in order to efficiently control the projection of the surface voxels, we do not use the projection with kernels to compute the boundaries. Instead, there are two projections computed at each gantry angle to extract the final 2D boundaries of the surface voxel projection. One is the projection with voxel kernels (similar to ordinary DRR computation) shown at the top right of Figure 8 and the other is without kernels (directly project each surface voxel onto the image plane) shown at the top left of Figure 8. We have to use the projections with kernels to compute rough DRRs (possibly aliasing exists, but we only care the image boundaries) and then employ it to filter out the exact 2D projection boundaries of the deformed anatomy surface shown at the bottom of Figure 9.

#### 2.3.3. Optimization of Boundary-Based 3D-2D DIR Energy

The computed projections of 3D surface are compared with corresponding 2D projection boundaries from CBCT scans, and the primary DVF is iteratively optimized to obtain a good initial deformation for final volumetric image. The surface deformation is optimized by minimizing the total energy *E*_bound_, which includes two terms, the regularization used to achieve smoothness of the DVF and the similarity between the two images that is different from the previous intensity-based formulation. Here, the projections of the anatomy surface voxel are compared with corresponding 2D projection boundaries from CBCT scans:

(19) Ebound=μLD+∑m=1NpdistminRboundD,θm,Iboundθm2,

where *μ* is a weighting factor and empirically set at 10.0 for the experiments. **D** is the DVF defined on the mesh vertices. *L*(**D**) is regularization term defined as in (10). The second term of the energy function indicates the similarity between the CBCT projections boundaries *I*_bound_(*θ*_*m*_) acquired at gantry angle *θ*_*m*_ beforehand and the projections *R*_bound_(**D**, *θ*_*m*_) created after the deformation of **D** applied on planning CT body surface at the same gantry angle. *N*_*p*_ is the number of projections involved. This term is a summation of the square of the shortest Euclidean distance between every pixel on projections of the deformed CT surface and the corresponding CBCT projection boundaries. *k*-*d* tree data structure is applied to efficiently search such nearest pixels for boundaries [38].

L-BFGS algorithm is used to optimize the DVF (**D**). The gradient of the energy function *E*_bound_ with respect to **D** can be calculated as follows:

(20) ∇Ebound=μ∇LD+2∑m=1NpdistminRboundD,θm,Iboundθm∇distminRboundD,θm,Iboundθm.

∇dist_min_(*R*_bound_(**D**, *θ*_*m*_), *I*_bound_(*θ*_*m*_)) can be computed numerically by finite difference with a small Δ**D**.

Because we do not need the exact DRRs of the deformed 3D anatomy image, the resampling is not required. What we focus on is the updated voxel positions of the deformed 3D anatomy surface. Then we can use the splatting method to compute the projections of the deformed volume surface.

After the above boundary-based registration, the primary DVF is obtained and then applied in further complete intensity-based DIR as the initial deformation. As a result, the final volumetric images are obtained by applying the optimized DVF to planning CT images.

## 3. Results

The algorithms are implemented by using Microsoft Visual C++ 2010, MATLAB R2013a, and NVIDIA CUDA 5.5. For the hardware platform, the experiments are run on a desktop computer with Intel® Xeon E5645 CPU with 2.40 GHz, 34 GB DDR3 RAM, and NVIDIA GeForce GTX 780 Ti GPU with 3 GB GDDR5 video memory.

We evaluate and compare our proposed nonuniform tetrahedral meshing for 3D-2D DIR with other 3D-2D DIR methods, that is, voxel-based method [1–3], uniform orthogonal grid mesh [16], and uniform tetrahedral mesh on image visualization and quantitative evaluations on two XCAT phantoms and five H&N cancer patients.

### 3.1. Evaluation

This method is evaluated thoroughly by using two sets of digital XCAT phantoms and H&N patient data. Taking the XCAT male phantom data for example, two sets of 3D images, representing the same patient (phantom) at two different respiratory phases, are created. Both the beating heart and respiratory motions are considered, and in order to simulate the large deformation, the max diaphragm motion is set to 10 cm. Phase 1 and Phase 4 are shown in Figures 9(a) and 9(b). Phase 1 data is used as the original planning CT image, while Phase 4 data represents daily CBCT images. The deformation vector of every voxel between these two phases is provided by the XCAT software and it is used as the ground truth of DVF for evaluation. A set of DRRs from Phase 4 data is created using a ray tracing method with noise simulation as mentioned in Section 2.2.2 and subsequently used as the raw projections from the daily CBCT. The original CT (Phase 1 data) is deformed to fit the raw projections of daily CBCT and finally a new set of volumetric images is created and compared with the ground truth, Phase 4 data. Simultaneously, the final DVF is compared with the DVF obtained from the XCAT software.

A conventional normalized cross correlation (NCC) is used to evaluate the similarity (i.e., the linear correlation) of 3D images and DVFs:

(21) $$\begin{array}{c} \text{NCC}=\frac{\sum_{i=1}^{N}\left[F_{intp}\left(i\right)-{\overset{-}{F}}_{intp}\right]\left[F\left(i\right)-\overset{-}{F}\right]}{\sqrt{\sum_{i=1}^{N}{\left[F_{intp}\left(i\right)-{\overset{-}{F}}_{intp}\right]}^{2}}\sqrt{\sum_{i=1}^{N}{\left[F\left(i\right)-\overset{-}{F}\right]}^{2}}}, \end{array}$$

where *F*_intp_(*i*) and *F*(*i*) are the interpolated and target values, respectively, over *N* voxels. ${\overset{-}{F}}_{intp}$ and $\overset{-}{F}$ are the average values of the interpolated and target values. The range of the NCC is [−1,1]. If NCC is 1, it means two values are exactly the same. The larger the NCC is, the more similar the values are.

The normalized root mean square error (NRMSE) between the interpolated values *F*_intp_(*i*) and the target values *F*(*i*) is also used for comparison of 3D images and DVFs and it denotes the related error:

(22) $$\begin{array}{c} \text{NRMSE}=\sqrt{\frac{\sum_{i=1}^{N}{\left[F_{intp}\left(i\right)-F\left(i\right)\right]}^{2}}{\sum_{i=1}^{N}{\left[F\left(i\right)\right]}^{2}}}. \end{array}$$

The range of the NRMSE is [0, +*∞*). If NRMSE is 0, it means two values are exactly the same. The smaller the NRMSE is, the more similar the values are.

**Figure 10:**
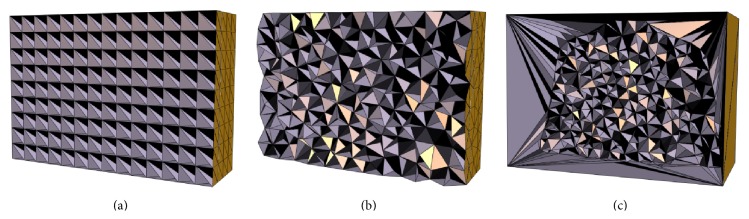
Three types of meshing for XCAT male phantom data. (a) A uniform orthogonal grid; (b) a uniform tetrahedron mesh; (c) a feature-based nonuniform tetrahedron mesh.

To demonstrate the advantage of using feature-based nonuniform mesh, results of three types of meshing methods are compared. These are (1) a bounding box uniform orthogonal grid in Figure 10(a), as mentioned in [9]; (2) a uniform tetrahedron mesh in Figure 10(b); (3) a feature-based nonuniform tetrahedron mesh in Figure 10(c). A voxel-based deformation method is also evaluated.

### 3.2. Meshing Computation

In order to apply the meshing-based method in the 3D-2D image registration framework, we have to compute the meshes at first. The number of vertices of the tetrahedron meshes in XCAT phantom data and H&N patient data are all around 1,000; hence the execution time of the particle-based meshing method is the same. H&N patient data (512 × 512 × 140) is larger than XCAT data (256 × 256 × 132), so it takes more time in the preprocessing steps of image feature edges computation and the density field computation for feature-based tetrahedron mesh generation. Compared with the uniform orthogonal grid mesh generation (5 seconds), the isotropic tetrahedron mesh generation needs 10 seconds, and the feature-based tetrahedron mesh generation needs more time in preprocessing steps: (a) compute the image feature edges: 3.5 seconds (XCAT data) versus 15 seconds (H&N patient data); (b) compute the density field: 0.4 minutes (XCAT data) versus 1.5 minutes (H&N patient data); (c) run particle-based meshing framework: 10 seconds. Uniform and feature-based nonuniform tetrahedron meshes can be generated by our particle-based meshing approach only if the desired density field is available (the density field of uniform tetrahedron mesh is globally uniform). Once these meshes are generated, they are used by the 3D-2D DIR framework, and no additional computation is required for the meshes. It should be noted that the mesh generation could be done in advance as soon as the planning CT is performed. So the time for this preprocess can be hidden for the image registration process.

### 3.3. XCAT Phantom Data

Feature-based nonuniform tetrahedral meshes are created using approximately 1,000 vertices on the original CT image (Figure 9(a), Phase 1 of the XCAT male model). 60 DRRs are created from the target image (Figure 9(b), Phase 4) at 60 different gantry angles equally spaced over 360 degrees. The new volumetric images are obtained by optimizing the deformation of the meshes by comparing the 60 DRRs of the deformed images with the corresponding projections of the target images in 100 iterations of the elastic registration algorithm. Figure 9(c) shows that the new reconstructed images are very close to the target images. Their differences are illustrated in Figure 9(d), which are very small.

**Figure 11:**
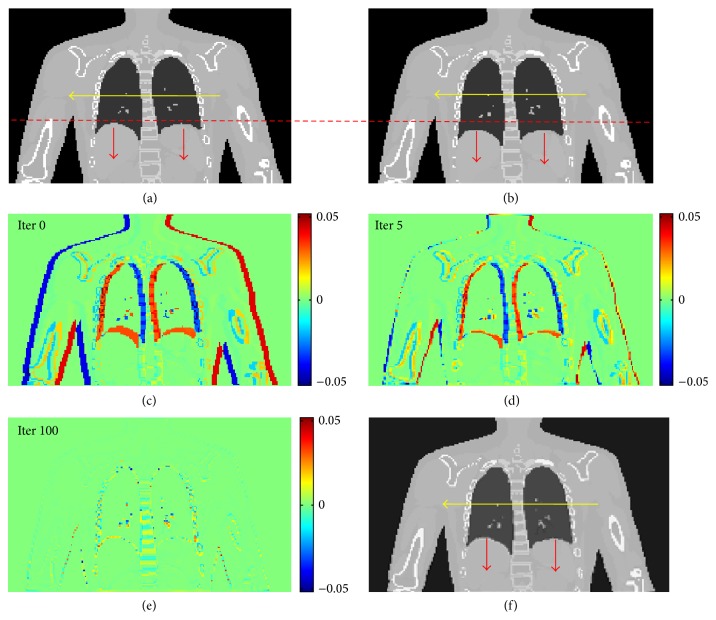
Demonstration of XCAT female phantom results. (a) Original image (Phase 1); (b) target image (Phase 4); (c) differences between deformed and target images at the beginning of optimization; (d) differences between deformed and target images at iteration 5; (e) differences between deformed and target images at iteration 100 (end of the optimization); (f) final deformed image from Phase 1.

To test the robustness and accuracy of the algorithm, another XCAT female phantom with more complicated motions including a large deformation between respiratory Phase 1 and Phase 4 and the translation (globally translated by four-voxel-size distance (i.e., about 4.68 mm) in the horizontal direction) is used. Other configurations are the same as the previous male phantom case.  Figure 11 shows the intermediate and final results during the optimization process.

While a conventional CBCT reconstruction requires hundreds of projections, the mesh-based algorithm uses far fewer projections since the information from the planning CT image is used. The number of projections may vary from case to case.  Table 1 lists the deformation results using varying numbers of projections. A larger number of projections do yield a higher NCC and lower NRMSE, though at the expense of longer calculation time and more radiation dose. A reasonable balance can be observed at 60 projections, as diminishing returns start to take effect by using a larger number of projections.

**Figure 12:**
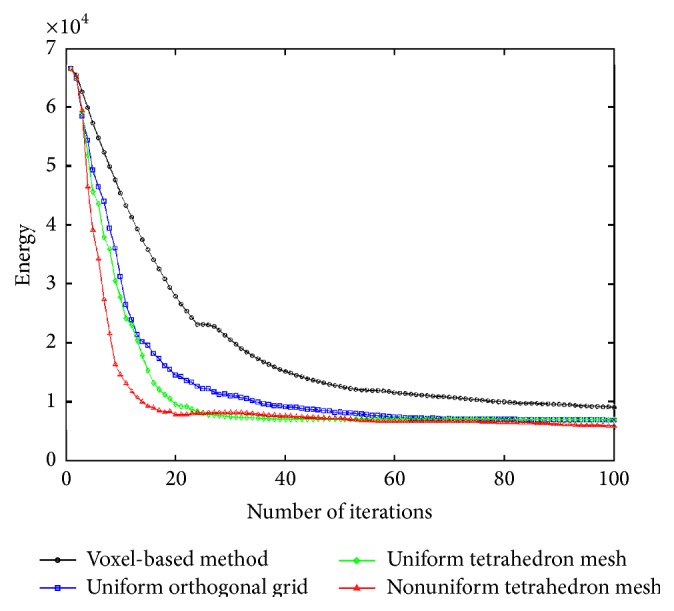
The similarity energy (*E*_sim_(**D**) term in (9)) curves of different methods (voxel-based, uniform orthogonal grid, uniform tetrahedron mesh, and nonuniform tetrahedron mesh) in image registration.

Comparisons of results from different meshing algorithms are shown in Tables 2 and 3. With regard to the similarity of the final images, the nonuniform mesh provides better results than the uniform orthogonal grid or uniform tetrahedron meshes in 100 optimization iterations, for the approximately same number of vertices. The uniform orthogonal grid mesh is the same as mentioned by Foteinos et al., who pointed out that it has the best registration performance in their experiments [16]. With more vertices, the nonuniform meshing method provides image results very close to the voxel-based method, which has as many vertices as voxels. It shows that the voxel-based deformation may yield good image intensity result, but the resulting DVF represents an unrealistic anatomical mapping. This is a drawback of voxel-based deformation and is due to its localized deformation (i.e., there are too many sampling points and it is easy to be trapped into some local minima). The feature-based nonuniform meshing method overcomes this drawback and yields more anatomically accurate DVF (both the NCC and NRMSE measurements on DVF of the nonuniform meshing method are better than those of the voxel-based method, which were applied in [1–3]). Furthermore, from Figure 12, it is clearly seen that the energy curve of the feature-based meshing method (the red line with triangle marker) decreases dramatically faster than any other methods; that is, it has faster convergence speed during the image registration optimization. It is noted that because different meshes or voxel-based methods may have different regularization terms, such as different numbers of displacement vectors, we only compare the similarity term in (9) to make the comparison fair. With the large translation in the XCAT female phantom, the results (in Table 3) of voxel-based method are not as good as the meshed-based methods in both images and DVF measurements, due to its localized deformation and translation.

### 3.4. H&N Patient Data

**Figure 13:**
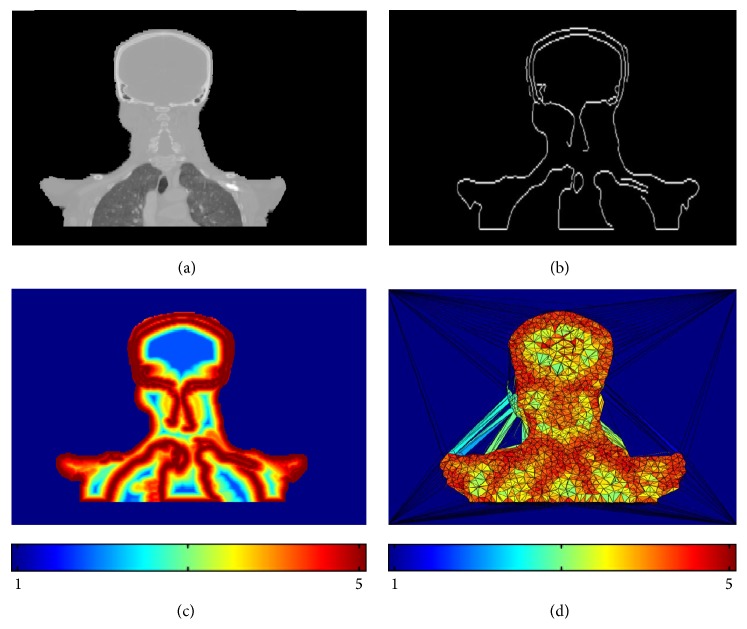
Demonstration of the feature-based mesh generation on the H&N01 patient data. (a) The original image; (b) extracted feature edges; (c) density field; (d) a 2D view of the interior meshes with color-mapping.

**Figure 14:**
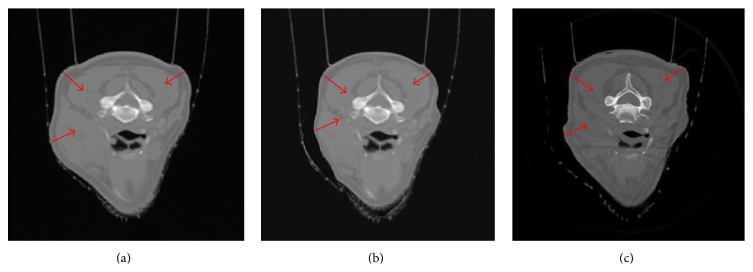
Demonstration of H&N01 patient results from axial view. (a) Original CT image; (b) deformed image; (c) target image (daily CBCT).

**Figure 15:**
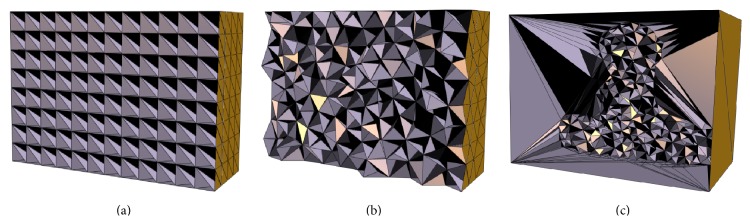
Three types of meshing for H&N01 cancer patient data. (a) A uniform orthogonal grid; (b) a uniform tetrahedron mesh; (c) a feature-based nonuniform tetrahedron mesh.

This feature-based nonuniform meshing image registration method has been tested on five clinical data sets from the head and neck cancer patients H&N01~H&N05.  Figure 13 illustrates the density field mapping based on image feature edges and feature-based tetrahedron mesh of H&N01 patient data.  Figure 14 shows the deformation results from axial view (a big tumor on the right side of the patient's chin shrinks) compared with the conventional CBCT reconstruction results of H&N01 patient data. The effects of various numbers of projections are also evaluated and the results are shown in Table 4. The three meshing methods, uniform orthogonal, uniform tetrahedron, and feature-based nonuniform tetrahedron (as shown in Figure 15), are evaluated with results shown in Table 5 for all patient data sets. The nonuniform meshing method again yields the highest accuracy and has faster convergence speed during the image registration. It is noted that, comparing the accuracies in the XCAT phantom data and the H&N patient data, the NRMSE seems larger in the patient cases (~0.5) than that in the XCAT cases (<0.2); this is because, in the patient case, the reconstructed CBCT image is computed based on the planning CT image, and the patient had the planning CT scan and daily CBCT scans on different days and machines, which may cause the differences in the image background, noises, and so forth, resulting in larger NRMSE values. However, it is acceptable in the image results as shown in Figure 14.

### 3.5. Boundary-Based 3D-2D DIR Results

**Figure 16:**
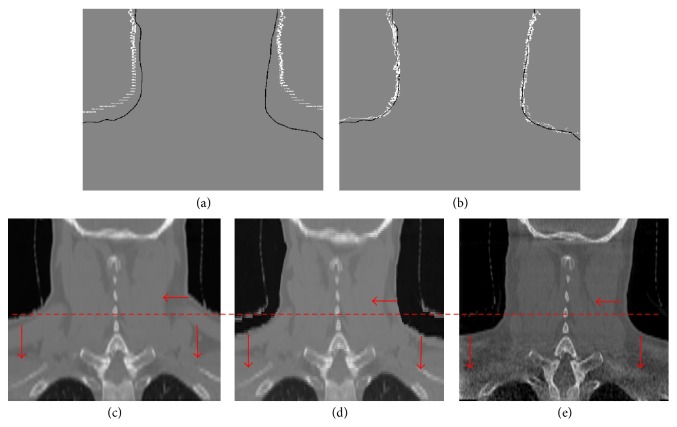
Demonstration of H&N05 patient data results. (a), (b) Differences between projection of the original/final surface voxel (white dots) and the target CBCT projection boundary (black dots); (c) original CT image; (d) reconstructed image from original CT image; (e) target image (daily CBCT).

For the H&N05 cancer patient data, which has a large deformation on tumor during treatment, we use it to evaluate the effectiveness of the boundary-based 3D-2D DIR method. Figures 16(a) and 16(b) show the original and final differences of one projection boundary in boundary-based DIR.  Figure 16(d) demonstrates that the final deformed CT images are very close to the target images (Figure 16(e)) and their differences are very small by further intensity-based 3D-2D DIR.

The accuracy of the boundary-based 3D-2D DIR and further intensity-based 3D-2D DIR are performed on H&N05 cancer patient data. Both the NCC and NRMSE shown in Table 6 demonstrate that boundary-based DIR can provide a good initial guess of the deformation for the further intensity-based 3D-2D DIR; that is, after the boundary-based 3D-2D DIR, NCC and NRMSE are close to the final 3D-2D DIR accuracy results.

With the GPU-based implementation, taking this H&N cancer patient data for example, our boundary-guided method takes 5.26 seconds per iteration, which is about 10 times faster than the non-boundary-guided method (i.e., 59.75 seconds). The multiresolution scheme is used to further improve the speed on both boundary-based 3D-2D DIR and further intensity-based 3D-2D DIR. In the experiment, only the CBCT images are downsampled into different resolution levels (three levels for experiments on H&N05 patient data), from the coarsest level (CBCT projection: 256 × 192, time for boundary-based DIR: 1.37 seconds/iteration, and time for intensity-based DIR: 3.15 seconds/iteration) to the higher level (CBCT projection: 512 × 384, time for boundary-based DIR: 2.11 seconds/iteration, and time for intensity-based DIR: 15.01 seconds/iteration) and finally to the full resolution level (CBCT projection: 1024 × 768, time for boundary-based DIR: 5.26 seconds/iteration, and time for intensity-based DIR: 59.75 seconds/iteration). At the same time, the proposed method needs fewer optimization iterations (35 iterations compared with original 50 iterations). The significant advantage of this method is that, instead of registering the whole image, we just need to register the surface voxel and projection boundaries, which involves much fewer number of voxels and pixels. In addition, in the H&N patient data case, we only focus on the 3D surface and 2D projection boundaries, so that the effects of different image modalities will be ignored. By using both GPU implementation and multiresolution scheme, the volumetric image reconstruction of 512 × 512 × 140 H&N cancer patient can be accomplished within 3 minutes (compared with 6.9 minutes of the original 3D-2D DIR in Section 2.2.5), so that this boundary-based 3D-2D DIR method could be probably used in the clinically practical studies.

## 4. Conclusion and Future Work

The feature-based nonuniform meshing allows more sampling points to be placed in the important regions; thus the deformation can be controlled more precisely. With the approximately same numbers of sampling points (vertices), the feature-based nonuniform meshing method produces better registration results, where a larger NCC is obtained compared with the uniform orthogonal grid and the uniform tetrahedron meshes. While this improvement may seem small, it is important to note that the NCC is very close to 1 because only minor anatomic changes occur. The NRMSE measurement is also provided to represent the differences between deformed images and the ground truth images. In H&N patient data, again, the feature-based nonuniform meshing method yields the highest accuracy of registration among the various methods.

It is intuitive that more sampling points (10,000 versus 1,000) lead to better results. In contrast, the voxel-based deformation provides the best image results in XCAT male phantom but requires using more than eight million sampling points. However, when the optimized DVF is compared with the ground truth DVF, the DVF of voxel-based deformation is significantly less similar to the ground truth than the feature-based nonuniform meshing method. Voxel-based deformation may yield better image intensity result, but the resulting DVF represents an unrealistic anatomical mapping. This is a drawback of voxel-based deformation and is due to its localized deformation. The feature-based meshing method overcomes this drawback and yields more anatomically accurate DVF.

As for the mesh quality, before deformation, the nonuniform tetrahedral meshes are generated based on the smooth density field as introduced in Section 2.1.2 so that we can obtain the high-quality adaptive tetrahedral meshes without any degradation and self-intersection. After image registration (deformation), the adaptive tetrahedral meshes are all good as well. These are two factors to guarantee no degraded and self-intersected tetrahedrons in the deformed meshes: (1) in the proposed energy function in (9), the regularization term *L*(**D**) and weighting factor *μ* are to achieve the smoothness of the DVFs. (2) The target DVFs are quite smooth in both the XCAT phantoms and H&N cancer patients. Of course, if the DVFs are highly varying with sharp discontinuities or weighting factor *μ* is not set properly, the deformed tetrahedral meshes will have problems, such as degradations or self-intersections. Since the mesh quality study is beyond the scope of this work, future studies are warranted.

The repeated use of CBCT during a course of treatment could deliver high extra imaging dose to patients. For example, if weekly CBCT pelvis scans are performed with the conventional faction scheme, the total dose will be around 4.05 mSv/scan × 6 weeks = 24.3 mSV; and the total dose of head scans will be around 2.0 mSv/scan × 6 weeks = 12.0 mSV (Table II in [39]). If the daily CBCT is performed, the total dose will be much higher. Using this method, far fewer projections are needed to produce a set of high-quality volumetric deformed images than in conventional CBCT reconstruction, which can dramatically reduce the radiation dose during CBCT scans. Additionally, it is clearly seen that the feature-based nonuniform meshing method has faster convergence speed than other methods during the registration process.

Moreover, the proposed boundary-based 3D-2D DIR method can substantially further improve both the accuracy and the speed of reconstructing volumetric images by producing a good initial DVF. This eventually will lead to a fast and safe daily volumetric imaging with a very small number of projections for image-guided radiation therapy or online adaptive radiation therapy. There might be a limitation of this boundary-based method, if the deformation happens mainly in the internal organs. In the case of lung, its intensity is significantly different from that of chest wall, so we may segment lung and apply the proposed boundary-based 3D-2D DIR method only focusing on the lung first.

In the future, our feature-based nonuniform meshing method may also be applied to 4D images registration. A CBCT scan acquires approximately 600 hundred projections in a full rotation, and if they are sorted into ten respiratory phases, the corresponding 4D simulation CT set can be used to generate a high-quality, full 4D CBCT image set without exposing the patient to additional imaging dose. Currently, the feature-based nonuniform meshing method has been employed to some head and neck cancer patient data and achieved excellent results. In the future, we will investigate and determine the clinical accuracy of the method based on more patient data and statistical analysis in some follow-up applications for other cancer cases: such as breast, lung, and prostate cancers.

## Figures and Tables

**Algorithm 1:**
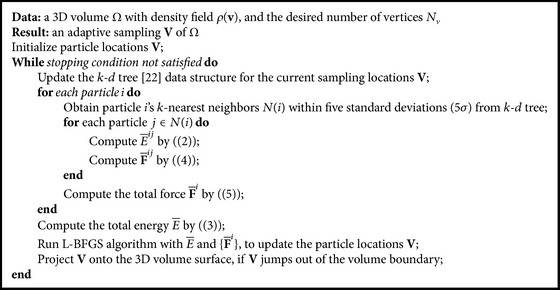
Particle optimization with density field *ρ*(**v**).

**Table 1:** Digital XCAT male phantom study results using various numbers of projections.

Number of projections used	10	20	30	60	90	120
NCC of images	0.9788	0.9812	0.9825	0.9855	0.9859	0.9860
NRMSE of images	0.1952	0.1836	0.1769	0.1612	0.1587	0.1585
NCC of DVF	0.7823	0.7946	0.8012	0.8150	0.8189	0.8201
NRMSE of DVF	1.0055	0.9076	0.8525	0.8118	0.7959	0.7951

*Note*. These comparison experiments are run through 100 iterations.

**Table 2:** Evaluation of reconstruction accuracy based on a digital XCAT male phantom.

	Uniform orthogonal grid	Uniform tetrahedron mesh	Nonuniform tetrahedron mesh	Nonuniform tetrahedron mesh	Voxel-based method
Number of vertices	1,050	987	1,005	10,004	8,650,762
NCC of images	0.9829	0.9835	0.9855	0.9858	0.9872
NRMSE of images	0.1749	0.1705	0.1612	0.1593	0.1514
NCC of DVF	0.7690	0.7829	0.8150	0.8265	0.6061
NRMSE of DVF	1.2366	1.0923	0.8118	0.8059	1.2484

*Note*. The three mesh-based methods are run through 100 iterations, while the voxel-based method needs 200 iterations.

**Table 3:** Evaluation of reconstruction accuracy based on a digital XCAT female phantom.

	Uniform orthogonal grid	Uniform tetrahedron mesh	Nonuniform tetrahedron mesh	Nonuniform tetrahedron mesh	Voxel-based method
Number of vertices	980	981	1,011	10,000	8,650,752
NCC of images	0.9789	0.9792	0.9829	0.9846	0.9775
NRMSE of images	0.1970	0.1954	0.1766	0.1682	0.2035
NCC of DVF	0.7687	0.7680	0.7672	0.7649	0.6292
NRMSE of DVF	1.3875	1.1358	0.9705	0.9777	1.0317

*Note*. The three mesh-based methods are run through 100 iterations, while the voxel-based method needs 200 iterations.

**Table 4:** Results with different numbers of projections on H&N01 patient data.

Number of projections used	10	20	30	60	90
NCC of images	0.8458	0.8459	0.8460	0.8460	0.8460
NRMSE of images	0.4798	0.4794	0.4792	0.4792	0.4792

*Note*. These comparison experiments are run through 50 iterations.

**Table 5:** Comparison of three meshes on the data of five head and neck cancer patients.

Patients	Uniform orthogonal grid	Uniform tetrahedron mesh	Nonuniform tetrahedron mesh
H&N01			
Number of vertices	936	992	1,007
NCC	0.8327	0.8358	0.8460
NRMSE	0.4988	0.4938	0.4792
H&N02			
Number of vertices	1,040	990	1,000
NCC	0.9036	0.9122	0.9134
NRMSE	0.4182	0.4116	0.4084
H&N03			
Number of vertices	980	1,000	998
NCC	0.8470	0.8471	0.8482
NRMSE	0.4748	0.4743	0.4722
H&N04			
Number of vertices	1,001	1,000	1,000
NCC	0.7756	0.7806	0.8111
NRMSE	0.5841	0.5817	0.5567
H&N05			
Number of vertices	980	1,000	995
NCC	0.7565	0.7690	0.7843
NRMSE	0.6278	0.6092	0.5712

*Note*. The three mesh-based methods are run through 50 iterations.

**Table 6:** Evaluation of boundary-based DIR accuracy on an H&N cancer patient data.

Status	Initial	Boundary-based DIR	Full DIR
NCC of images	0.7627	0.7875	0.7919
NRMSE of images	0.5938	0.5569	0.5479
